# Toward better drivability: Investigating user preferences for tip-in acceleration profiles in electric vehicles

**DOI:** 10.1371/journal.pone.0311504

**Published:** 2024-12-12

**Authors:** Seonghyeon Kim, Jaesik Yang

**Affiliations:** 1 Faculty of Electrical and Computer Engineering, Institute of Acoustics and Speech Communication, Chair of Acoustics and Haptics, Technische Universität Dresden, Dresden, Germany; 2 Institute of Advanced Technology Development, Hyundai Motor Company, Seongnam-si, Gyeonggi-do, South Korea; 3 Department of Industrial Engineering, Seoul National University, Seoul, South Korea; Chennai Institute of Technology, INDIA

## Abstract

Vehicle drivability, defined as the smooth operation and stability of a vehicle in response to driver inputs, significantly influences the performance of passenger cars. Among various driving conditions, tip-in acceleration is one of the most frequently encountered and crucial factors affecting drivability. This study investigates preferred longitudinal acceleration profiles for electric vehicles through subjective evaluations obtained from on-road tests. Five distinctive acceleration profiles were designed for tip-in conditions and evaluated by 15 highly experienced experts using the paired comparison method. Evaluations were conducted across three scenarios: light tip-in at 30 km/h, middle tip-in at 30 km/h, and middle tip-in at 60 km/h. Experimental results revealed distinct preferences based on driving conditions. For light tip-in at 30 km/h, drivers favored linear acceleration profiles with smaller jerk magnitudes and kurtosis. Conversely, for middle tip-in conditions at both 30 and 60 km/h, drivers preferred acceleration profiles exhibiting rapid initial acceleration followed by a smooth transition. However, at 60 km/h, a preference for higher jerk and steeper gradients was observed. Correlation analyses provided insights into the relationship between subjective preferences and dynamic characteristics of acceleration profiles. This study contributes practical guidance for designing optimal acceleration profiles aligned with driver preferences, thereby enhancing drivability and overall user experience in electric vehicles.

## 1. Introduction

Vehicle drivability is a fundamental factor that significantly influences the performance of passenger cars [1,2]. Drivability refers to the smooth operation and stability of vehicles while the driver controls the powertrain under various driving conditions [3]. In general, drivability is determined by how well the vehicle responds to the driver’s intentions, such as acceleration, deceleration, and maintaining a constant speed. Consequently, drivability is developed and evaluated considering various driving conditions, including drive away, tip-in, tip-out, deceleration, and gear shifting [4]. In the automotive domain, "tip-in" refers to a maneuver characterized by a minor displacement of the accelerator pedal. Among these conditions, tip-in acceleration is the most frequently encountered and one of the most important factors [5], making it the focus of this study. This study specifically focuses on tip-in acceleration during cruising. During the vehicle development process, the design of the longitudinal acceleration profile for tip-in conditions necessitates careful consideration of multiple factors, including low torque at low engine speed, response delay, transient torque inaccuracies resulting from airflow control latency, acceleration lag, powertrain and transmission hesitation, and non-linear acceleration. Achieving an optimal tip-in longitudinal acceleration profile requires developer expertise based on empirical knowledge of vehicle dynamics [4]. Practical optimization involves conducting numerous experiments to minimize tip-in shock and refine acceleration shapes. Moreover, different engineers may propose varying acceleration profiles based on their experiences and tendencies. In practice, these proposed profiles undergo validation through user evaluations, leading to the derivation of the final acceleration profile. Consequently, developing the tip-in acceleration profile is a time-consuming process that necessitates an understanding of user subjective preferences.

Therefore, in order to improve efficiency in vehicle development, several quantitative studies [1,2,6–8] have been proposed to calibrate drivability. Dorey and Holmes reported that vehicle drivability is strongly influenced by the overshoot and rise rate of acceleration [1]. Moreover, Dorey and Martin developed the in-vehicle tools that can aid in the calibration of vehicle drivability [2]. They presented the capabilities and use of the proposed tool, as well as their functionality and examples of their use. Although they provided insight into the capabilities, functionality, and examples of use for this tool, they did not present specific results. Walters et al. [6] presented the methodology that can reduce the cost of vehicle drivability evaluation and powertrain refinement. They proposed the use of an analysis-based co-simulation methodology that uses powertrain simulation and objective drivability evaluation tools to predict vehicle drivability. Shin et al. [7] also presented the simulation method to evaluate the drivability considering the acceleration value, the vibration dose value, response delay and peak-to-peak value of the acceleration and its jerk. Oemler et al. [8] introduced the calibration tool that can compare the vehicle dynamic characteristics such as maximum acceleration level, response time, and jerk, to conduct the objective drivability analysis. These previous studies primarily focus on the calibration of dynamic attributes, such as acceleration, jerk, and delay optimization, through objective analysis of acceleration signals. However, a quantitative approach cannot provide direct insight into the specific acceleration patterns preferred by drivers. Therefore, subjective evaluation studies are essential for establishing development targets from a user perspective. Several studies have presented on subjective evaluations of longitudinal acceleration and drivability [9–12]. Chandrasekaran et al. [10] presented the result of objective and subjective evaluations for compact sport utility vehicles. They conducted the objective analysis using AVL-DRIVE and then analyzed the correlation for subjective evaluation conducted by expert drivers. Liu and Huang [11] proposed the fuzzy hierarchy quantization method to improve the subjective evaluation. They judged the drivability using the criteria such as acceleration response, transient performance, and ride comfort. Baumgartner et al. [12] presented a perceptual approach to evaluate drivability using a dynamic driving simulator. They reported the just noticeable difference for various acceleration levels. Their findings, however, were based on simulations rather than real-world driving conditions. They acknowledged that the sensory and dynamic feedback experienced by drivers in a simulator can differ from that in an actual vehicle, which could potentially affect the generalizability of the results to real-world conditions.

Meanwhile, electrified vehicles (EVs), including hybrid electric vehicles (HEVs) and battery electric vehicles (BEVs), exhibit superior control capabilities during longitudinal acceleration compared to internal combustion engine (ICE) vehicles. This is attributed to the rapid response characteristics of electric motors and their high torque output at low speeds. Consequently, the acceleration profile of EVs differs from that of ICE vehicles. Therefore, it is necessary to optimize the acceleration profile to align with the specific dynamic characteristics of EVs. Jauch et al. [13] presented a control architecture aimed at enhancing drivability in hybrid electric vehicles, with a focus on tip-in and tip-out acceleration response. They integrated a feedback controller with a disturbance observer loop and demonstrated significant improvements in drivability through simulations and experimental road tests. However, their work did not address the subjective evaluation of drivability, and the experimental road tests were limited in scope, potentially failing to cover all possible real-world driving conditions or vehicle models. Eller et al. [14] developed a co-simulation platform to optimize drivability in electric vehicles. Their study emphasized the importance of adjusting software and hardware parameters early in the design process to enhance drivability by adjusting torque setpoints and reducing shock amplitudes during tip-in maneuvers. Nevertheless, the co-simulation platform heavily relies on software models, which might not accurately capture the physical complexities of electric vehicles or account for all real-world variabilities and driver behaviors. Stoica et al. [15] investigated drivability issues in EVs, focusing on dynamic transient responses during tip-in maneuvers. Their work involved testing different models of electric vehicles to identify the most suitable one for drivability studies. Although they conducted practical evaluations of various mid-size vehicles, their focus was relatively narrow, concentrating on model selection for drivability studies without extending to comprehensive validation with empirical data. Consequently, there was a lack of integration of the results into a practical context of target setting, which might limit the applicability of the findings. Dou et al. [16] described a control-oriented drivability model for electric vehicles, capable of predicting longitudinal vehicle responses. While their model is useful for the design and calibration of control algorithms, the validation of the model is restricted to specific simulation environments (Matlab Simulink and CRUISE), which may not represent real-world driving conditions. Nandi et al. [17] proposed a comfortable optimal driving strategy for EVs, utilizing multi-objective optimization techniques. Their research focused on the design of optimal acceleration profiles and total acceleration duration to enhance driving comfort. The study employed computational simulations, which demonstrated a system response time of approximately 1 second, suggesting the strategy’s potential suitability for real-time implementation. Moreover, Zhang et al. [18] proposed a control design approach for optimizing the comfort of HEVs in acceleration mode using a genetic algorithm to determine the optimal power distribution and gear shift strategy, significantly enhancing ride quality during acceleration. Fuse et al. [19] proposed a novel speed control methodology aimed at optimizing the longitudinal acceleration and deceleration patterns of EVs to enhance passenger ride comfort. They applied optimal control theory to evaluate acceleration variations and jerk, with the objective of improving overall ride quality. Numerical simulations demonstrated that the proposed method significantly enhanced ride comfort and vehicle operational safety compared to conventional control strategies. However, the aforementioned studies [17–19] are primarily based on simulations and may not fully represent real-world conditions. Additionally, these studies did not take into account subjective driver experiences or the variability of road conditions, thereby presenting certain limitations.

While numerous studies have investigated the drivability of electric vehicles, the preponderance of research has primarily focused on vehicle control strategies and performance enhancements [13,17–25]. Furthermore, drivability is considered as a critical constraint in the energy management of EVs, and a substantial body of research is directed towards addressing this aspect [26–30]. The optimization of acceleration profiles necessitates the incorporation of subjective evaluations from the user’s perspective, with a particular emphasis on drivability. However, a comprehensive review of the extant literature reveals a notable paucity of studies specifically investigating the preferred longitudinal acceleration profiles of electrified vehicles from a drivability standpoint. Moreover, there is a dearth of subjective studies based on actual vehicle tests. This lacuna in empirical research has resulted in the current reliance on a trial-and-error approach for the development of drivability in EVs.

To address the limitations of previous studies, this study aims to examine preferred longitudinal acceleration profiles for electric vehicles by utilizing subjective evaluations obtained from on-road tests. By offering design guidance for longitudinal acceleration profiles from a subjective standpoint, the findings of this study can enhance the effectiveness of drivability development strategies. To accomplish this objective, five distinctive acceleration profiles for tip-in conditions were devised and evaluated. The proposed acceleration profiles were assessed and ranked according to preference through real-world driving evaluations conducted by experienced evaluators. The preferred profiles were determined based on the outcomes of these subjective evaluations, and a subsequent analysis was conducted to elucidate the characteristics of these favored acceleration patterns. By leveraging subjective evaluations from on-road tests, this study aims to contribute to the field of drivability development by providing practical insights into the design of longitudinal acceleration profiles that align with driver preferences.

The structure of this paper is organized as follows: Section 2 provides a detailed description of the design concept employed in crafting the longitudinal acceleration profiles. The evaluation procedure, encompassing the methodology and participant details, is presented in Section 3. Section 4 presents the results of the subjective evaluations, including an analysis of the preferred acceleration profiles and their dynamic characteristics. Finally, Section 5 concludes this study by summarizing the key findings, highlighting their practical implications, and discussing potential avenues for future research.

## 2. Design of longitudinal acceleration profiles

### 2.1 Experimental scenarios

**Fig 1:**
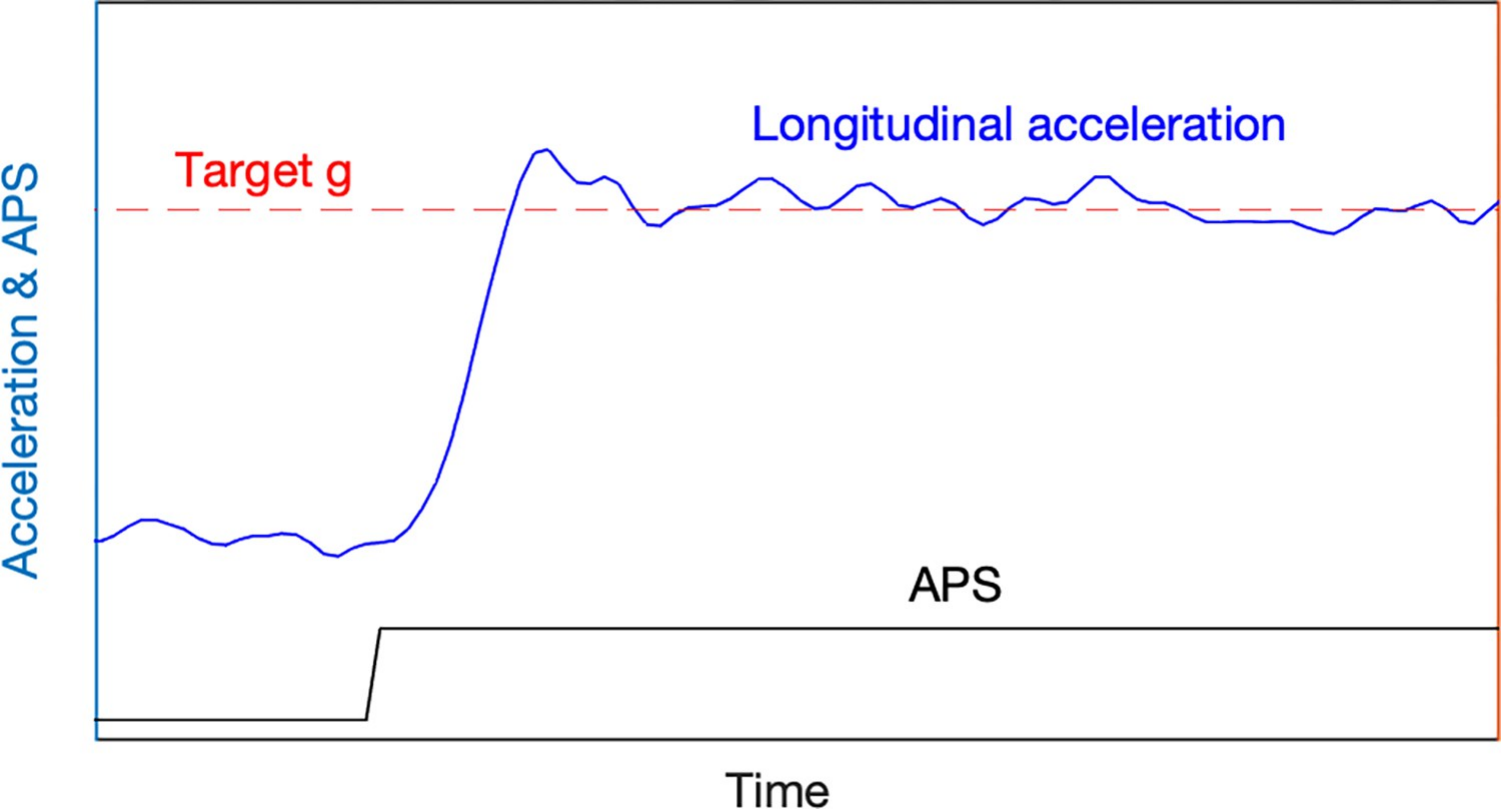
Representation of typical tip-in acceleration profiles.

Longitudinal tip-in acceleration profiles are typically designed by tuning the torque filter [31,32], as depicted in Fig 1. Torque filter tuning is a common approach employed to minimize the initial tip-in shock, allowing the target acceleration to be reached promptly while reducing the shock and jerk upon reaching the target. Tip-in acceleration can be categorized into three conditions based on the extent of accelerator pedal input: light tip-in (LTI) for slow acceleration, middle tip-in (MTI), and heavy tip-in (HTI). Generally, light tip-in corresponds to less than 30% accelerator pedal input, middle tip-in to 30–60%, and heavy tip-in to more than 60%. The driver’s intention to accelerate can be inferred from the degree of pedal depression.

This study aims to determine the preferred longitudinal acceleration profiles for common driving conditions. The experiment focused on two primary types of driving scenarios: light tip-in for slow acceleration and middle tip-in for moderate or rapid acceleration, which are commonly encountered in real-world driving situations. Three specific driving conditions were investigated: light tip-in at 30 km/h (Experiment 1), middle tip-in at 30 km/h (Experiment 2), and middle tip-in at 60 km/h (Experiment 3). To implement the longitudinal tip-in acceleration profiles, the torque filter was tuned based on the vehicle speed and accelerator pedal sensor (APS) readings for Experiments 1, 2, and 3. The driving conditions for Experiments 1–3 are summarized in Table 1.

**Table 1:** Experimental scenarios and their driving conditions.

Experimental condition	Initial vehicle speed (km/h)	Tip-in amount	Target acceleration (g)
**1**	30	Light	0.10
**2**	30	Middle	0.15
**3**	60	Middle	0.10

### 2.2 Design of longitudinal acceleration profiles

**Fig 2:**
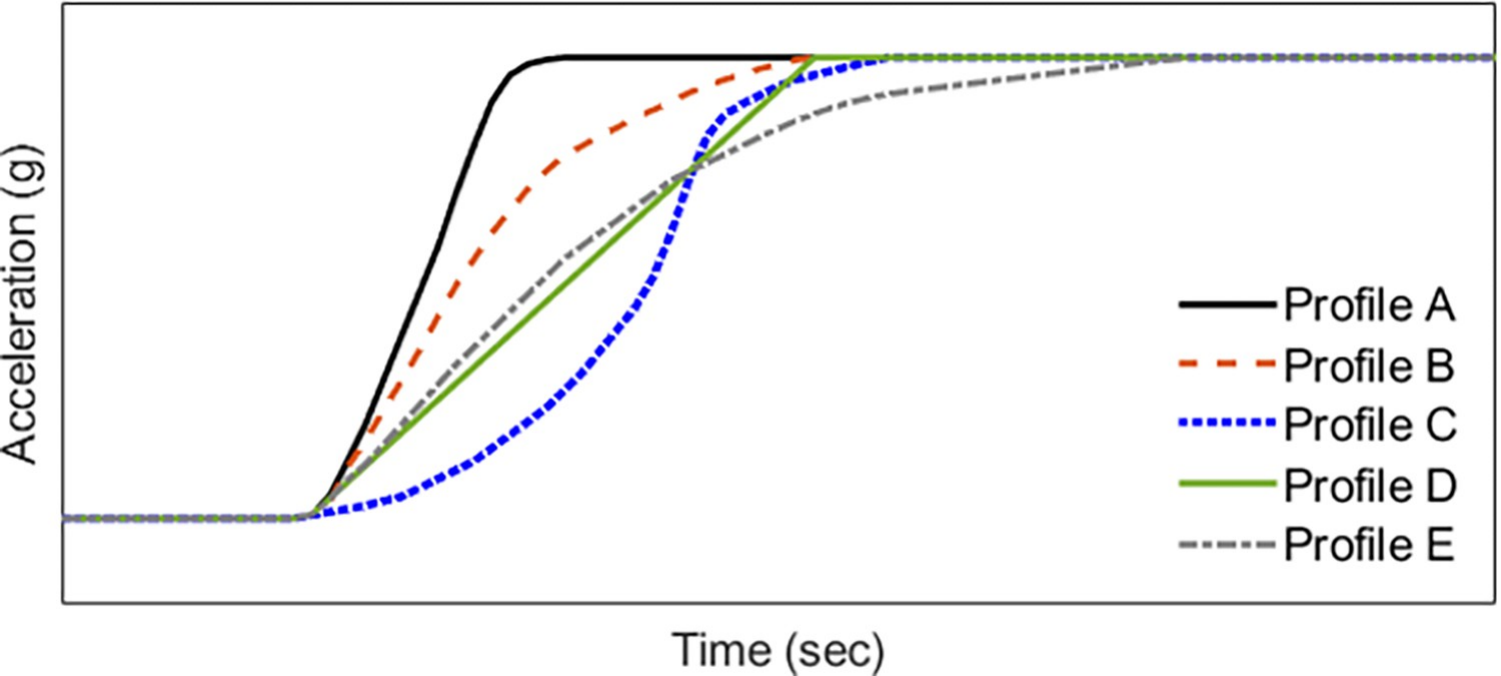
Designed longitudinal acceleration profiles.

Five representative acceleration profiles, designated as Profile A, B, C, D, and E, were designed for the experiments, as illustrated in Fig 2. Profile A represents the ideal acceleration slope, characterized by the fastest approach to the driver’s desired acceleration. Profile B is commonly employed as a benchmark for torque filter tuning, as it enables rapid initial acceleration followed by a smooth progression towards the target acceleration. Profile C is specifically designed to mitigate the shock caused by backlash during the initial acceleration phase. It features a slow initial acceleration that exceeds the speed at which backlash occurs, followed by rapid acceleration to smoothly reach the desired acceleration. Profile C is typically utilized when tuning vehicles with significant backlash. Profile D represents a linear acceleration curve toward the target acceleration value, with a similar time frame as Profiles B and C for reaching the target acceleration. Profile E exhibits similarities to Profile B, with an initial acceleration level comparable to Profile D. However, it demonstrates a relatively gradual rate of acceleration.

**Fig 3:**
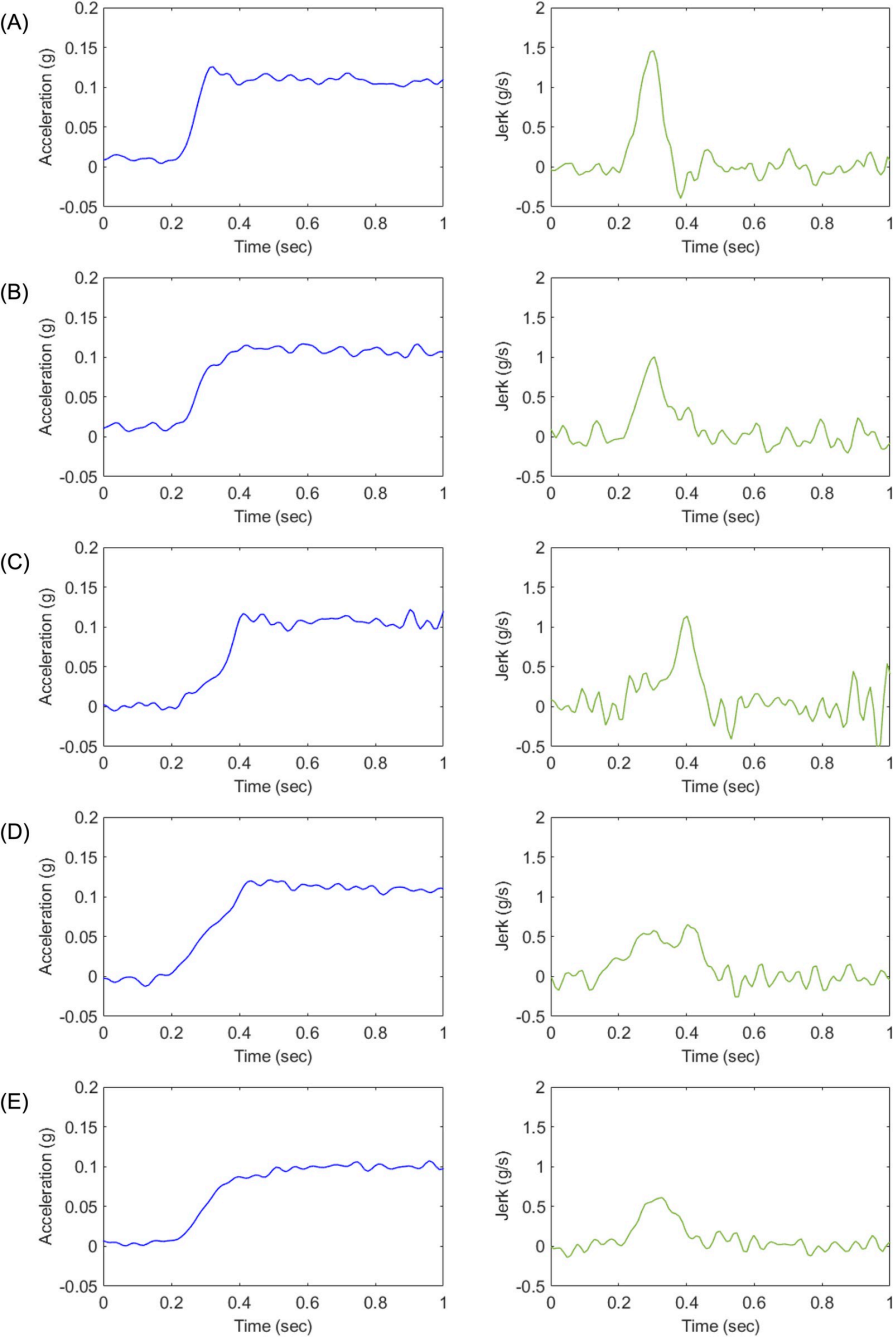
Longitudinal acceleration and jerk of each designed acceleration profile (A-E) for Experiment 1.

**Fig 4:**
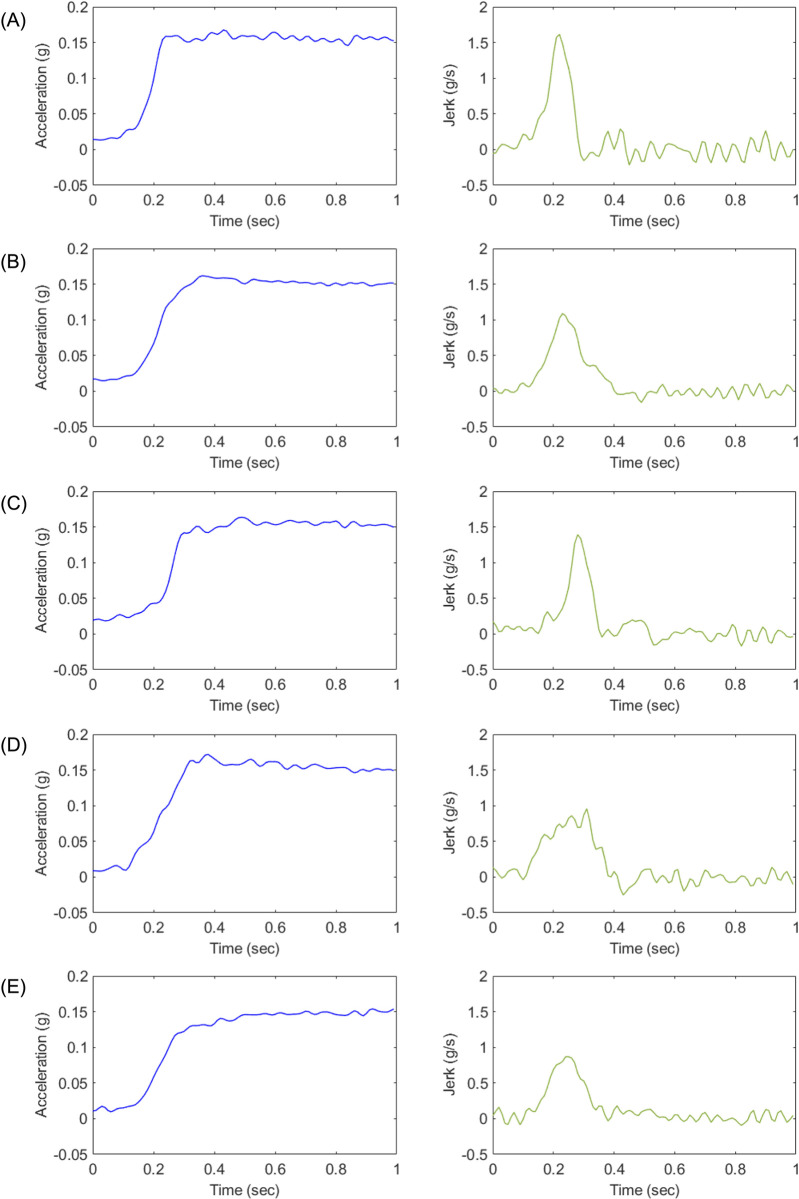
Longitudinal acceleration and jerk of each designed acceleration profile (A-E) for Experiment 2.

**Fig 5:**
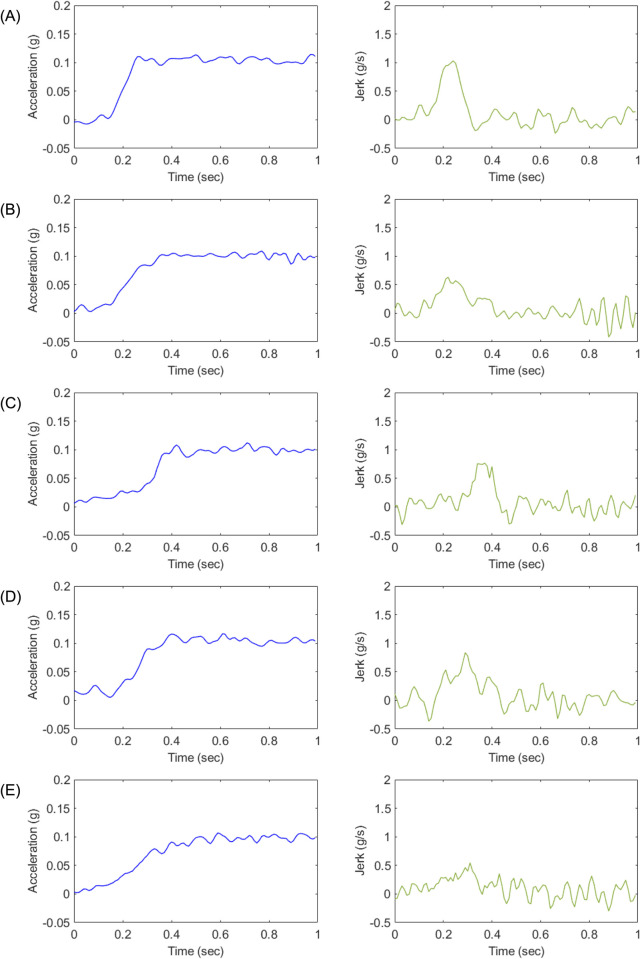
Longitudinal acceleration and jerk of each designed acceleration profile (A-E) for Experiment 3.

The acceleration profiles designed for each experimental case are presented in Figs 3 through 5. Fig 3 depicts the longitudinal acceleration and jerk profiles for Experiment 1, while Figs 4 and 5 illustrate the corresponding profiles for Experiments 2 and 3, respectively. To capture the dynamic behavior of the test vehicle during the experiments, various signals, including vehicle speed, motor speed, torque, acceleration, and APS, were measured using a high-speed controller area network system (CAN). The measurements were performed with a resolution of 10 milliseconds, taking into account the time taken from the start of the tip-in acceleration to reach the target acceleration, typically within a time frame of less than 0.5 seconds. Furthermore, the designed acceleration profiles were implemented in the engine management system engine control unit (EMS ECU), and a Hybrid Electric Vehicle (Elantra Hybrid 22MY, Hyundai) was utilized for the experiments. The experiments were conducted in the electric vehicle mode to evaluate the performance of the acceleration profiles.

## 3. Evaluation procedure

### 3.1 Subjects

The subjective evaluation involved the participation of fifteen highly experienced experts in the field of drivability development and assessment, all of whom were male. Most participants possessed more than a decade of experience in drivability development and had undergone extensive training in the sensitive assessment of longitudinal acceleration changes. The age range of the participants spanned from 31 to 55 years, with a median age of 37.0 years and a standard deviation of 6.4 years. The study was approved and conducted adherence to Hyundai Motor Company’s internal research protocols (reference number: HMC-IATD-2022-03-0002). Participants were voluntarily recruited through an advertisement, and the experimental phase was conducted from April 1, 2022, to April 30, 2022. Before the assessment commenced, participants were thoroughly informed about the purpose and procedures of the test, and their informed consent was obtained in accordance with the Declaration of Helsinki. Participants clearly understood the purpose and procedure of the test, subsequently submitted their written consent.

### 3.2 Evaluation procedure

**Fig 6:**
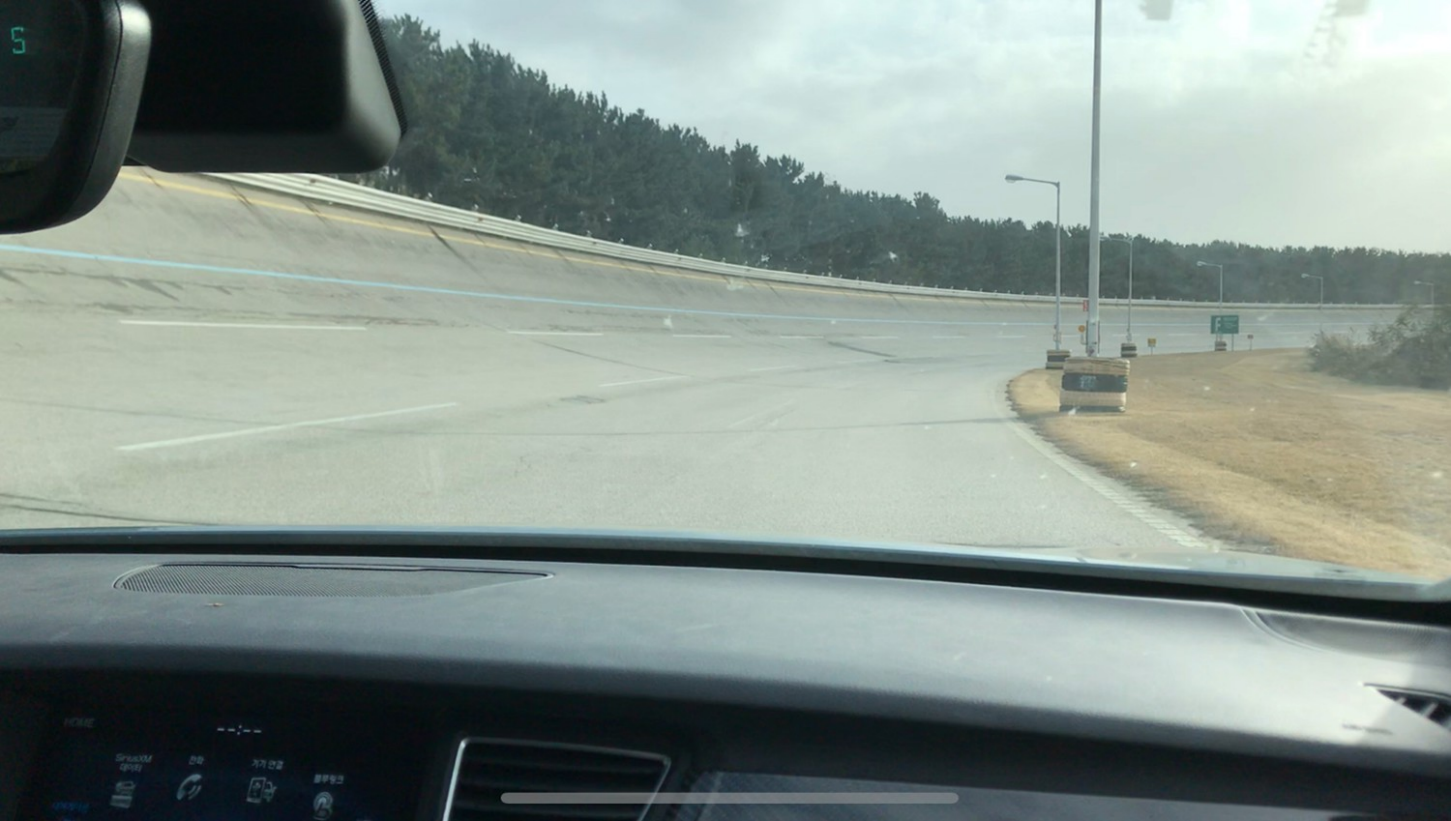
Test environment for the subjective evaluation.

The subjective evaluation was conducted through real-world vehicle operation at the proving ground of the Hyundai R&D Center (Hyundai Motor Company, South Korea). Fig 6 depicts the test environment. Since both the participants and researchers are internal employees, the experiments on the test track were conducted without the need for any permissions. The inherent subjectivity of expert evaluations can be acknowledged as a potential source of variability and bias in the results. To mitigate these risks and enhance the reliability of our findings, the following methodological measures were implemented:

- Evaluator selection: A panel of 15 evaluators was meticulously selected based on their extensive experience in drivability development, with each expert possessing a minimum of 10 years of relevant expertise. This stringent selection criterion is crucial, as laypersons may lack the capacity to discern subtle variations in acceleration profiles with the requisite precision. The involvement of these highly skilled professionals ensured that the evaluations were informed by a comprehensive and nuanced understanding of electric vehicle performance dynamics.
- Methodology selection: The paired comparison method was specifically employed to minimize cognitive load and mitigate individual bias. This approach necessitates direct comparisons between two acceleration profiles sequentially, rather than simultaneous evaluation of all profiles. Particularly in this study, where the differences in acceleration profiles between experimental cases were not significantly pronounced, the paired comparison method administered by expert evaluators was deemed an effective and practical approach.
- Perceptual calibration: Prior to the formal evaluation, all experts participated in a pre-driving calibration session. During this phase, evaluators were exposed to vehicles exhibiting each of the five distinct acceleration profiles to ensure their ability to discern the nuanced differences. This preliminary assessment confirmed the evaluators’ capacity to reliably perceive variations in acceleration profiles, thereby validating the subsequent evaluation process.
- Bias mitigation: During the training phase, evaluators were instructed to base their assessments on personal preferences rather than established vehicle development criteria. This approach aimed to address potential biases arising from ingrained development guidelines and to capture authentic user-centric perspectives.

These methodological considerations were implemented to enhance the robustness and validity of the study’s findings while acknowledging the inherent limitations of subjective expert evaluations in scientific research.

**Fig 7:**
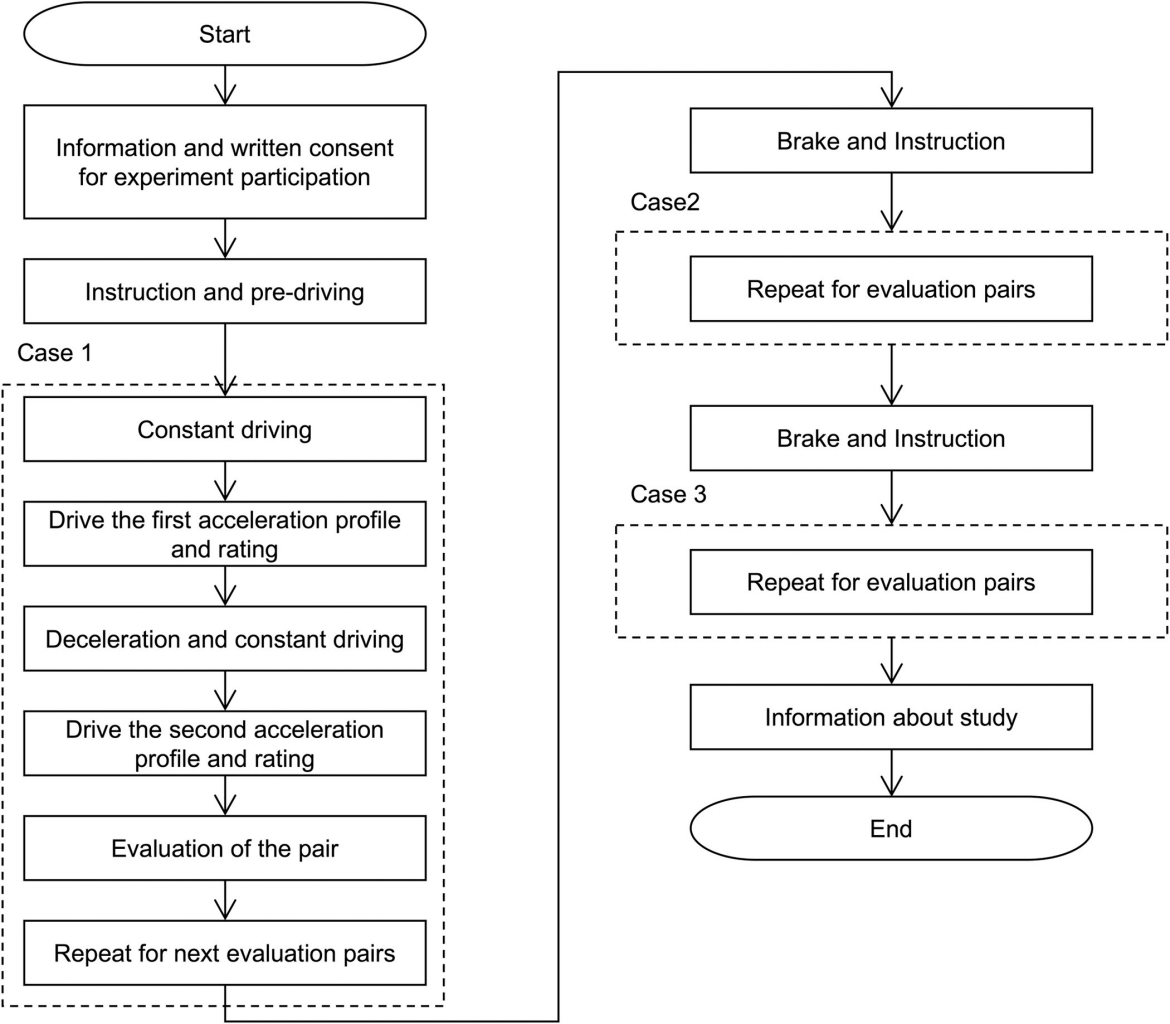
Subjective evaluation procedure.

In the implementation of the paired comparison method, a total of 10 evaluation pairs were systematically designed, adhering to the formula N(N-1)/2, where N represents the number of distinct acceleration profiles (N = 5). This methodological approach ensures a comprehensive comparison of all possible profile combinations. To reduce potential biases arising from learning effects or fatigue, each pair was evaluated in a randomized order. Fig 7 provides an overview of the evaluation procedure. Prior to the evaluation, participants engaged in sufficient pre-driving on the test track to familiarize themselves with the test environment. This involved maintaining a constant speed to stabilize the vehicle before initiating the tip-in acceleration. In Experiment 1, participants executed a light tip-in acceleration maneuver for approximately 5 seconds, utilizing the first acceleration profile of the designated evaluation pair, while maintaining a constant velocity of 30 km/h. Upon reaching the target acceleration value, participants decelerated the vehicle, evaluated the test scenes, and continued driving at a constant speed of 30 km/h. Subsequently, the light tip-in acceleration was conducted for the second acceleration profile of the evaluation pair, and participants selected their preferred acceleration profile from the two options. This evaluation process was repeated for subsequent experimental pairs. After completing the evaluation for Experiment 1, participants were afforded a short respite before proceeding to evaluate Experiment 2 and Experiment 3 using the same procedure.

Meanwhile, in our study, we aimed to isolate the effect of tip-in acceleration profiles by controlling other variables as much as possible. To manage these factors during the test, we implemented the following measures:

- Consistent road conditions: All trials were conducted on a designated test track with uniform road conditions to eliminate variability due to road surface differences.
- Vehicle load: The vehicle load was kept constant throughout all tests by ensuring the same number of passengers (the driver and one observer) and no additional cargo. This control helped to mitigate the impact of weight variations on acceleration performance.
- Environmental conditions: Tests were conducted under similar weather conditions to avoid the influence of external factors such as wind, temperature, and humidity on vehicle performance. Testing sessions were scheduled to avoid adverse weather conditions.
- Vehicle settings: The vehicle’s settings, including tire pressure, drive mode, and HVAC, were maintained consistently for all test runs to ensure that the mechanical conditions of the vehicle did not introduce variability.

By controlling these factors, we aimed to focus solely on the influence of the tip-in acceleration profiles on driver preferences. While real-world driving includes a broader range of variables, our controlled approach allows for a clearer understanding of the specific impact of tip-in acceleration profiles.

## 4. Experimental results

**Fig 8:**
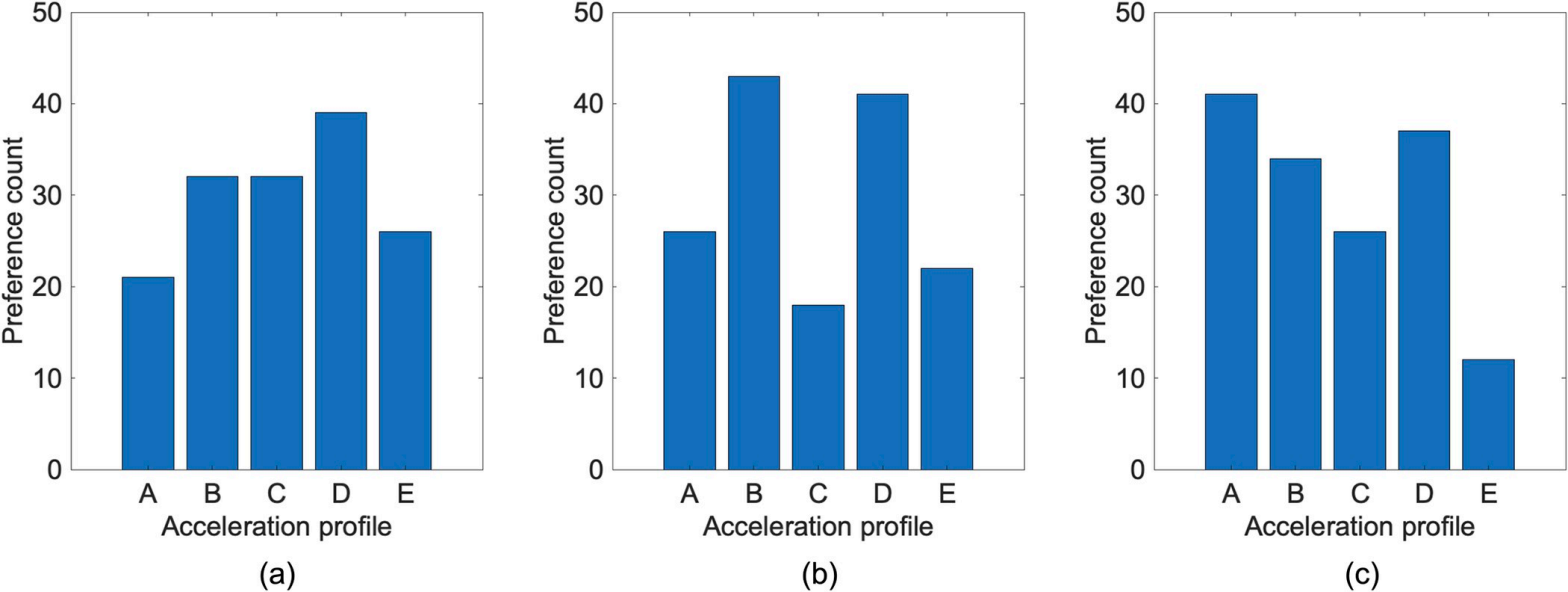
Preference counts for each acceleration profile in (a) Experiment 1, (b) Experiment 2, and (c) Experiment 3.

In the paired comparison method for the subjective evaluation, evaluators made direct comparisons between two acceleration profiles, and their preferences were recorded. For each experimental pair, we calculated the number of wins for each acceleration profile, which is represented in Fig 8.

In evaluating driver preferences for different longitudinal acceleration profiles, it is crucial to employ a robust and reliable method that accurately captures the relative strengths of each profile. Traditional ranking methods based on simple win counts, where each profile’s rank is determined by the number of times it is preferred over others, have significant limitations. These limitations include the inability to account for the relative strengths of competitors and potential biases introduced by an uneven distribution of comparisons. To overcome these challenges, we utilized the Bradley-Terry model, a probabilistic model specifically designed for paired comparison data [33]. This model offers several advantages over the simple win count method [34–37], making it more suitable for our study:

- Consideration of relative strength: The Bradley-Terry model estimates the probability that one profile is preferred over another, considering the relative strengths of all profiles involved. This ensures that the model accounts for the varying degrees of preference rather than just the frequency of wins.
- Handling uneven comparisons: In practical scenarios, it is often challenging to ensure that all profiles are compared an equal number of times. The Bradley-Terry model accommodates this unevenness by using maximum likelihood estimation to derive ability parameters for each profile. This results in more accurate and reliable rankings.
- Statistical significance: The model provides estimates along with their standard errors, enabling the calculation of confidence intervals. This statistical framework allows us to assess the significance of the differences between profiles, offering a deeper understanding of the data beyond mere win counts.
- Comprehensive analysis: The Bradley-Terry model integrates all available comparison data into a single framework, providing a holistic view of the preferences. This integration is particularly useful when dealing with complex datasets where direct ranking is not feasible.

By leveraging the Bradley-Terry model, we aim to provide a more nuanced and statistically robust analysis of driver preferences for longitudinal acceleration profiles. This method not only enhances the accuracy of our findings but also offers insights that would be missed by simpler ranking methods. The Bradley-Terry model is a probabilistic model used for analyzing paired comparison data. This model assumes that each item (in this case, each acceleration profile) has an underlying ability parameter that determines its likelihood of being preferred in a comparison. The probability that profile *i* is preferred over profile *j* is given by:

$$P(i\;beats\;j)=\frac{\theta_{i}}{\theta_{i}+\theta_{j}}$$ (1)

where *θ*_*i*_ and *θ*_*j*_ are the ability parameters for profiles *i* and *j*, respectively. These parameters are estimated using maximum likelihood estimation, which considers all pairwise comparisons and provides a comprehensive ranking based on relative strengths [34]. The likelihood function *L* for a set of pairwise comparisons can be expressed as:

$$L(\theta )=\prod_{i\neq j}{\left(\frac{\theta_{i}}{\theta_{i}+\theta_{j}}\right)}^{y_{ij}}{\left(\frac{\theta_{j}}{\theta_{i}+\theta_{j}}\right)}^{{n_{ij}-y}_{ij}}$$ (2)

where *n*_*ij*_ represents the number of times profile *i* has been compared to profile *j*, and *y*_*ij*_ denotes the number of times profile *i* defeated profile *j*. To facilitate the maximization process, we take the natural logarithm of the likelihood function to obtain the log-likelihood function ℓ(*θ*):

$$l(\theta )=\ln\;L(\theta )=\sum_{i\neq j}\left[y_{ij}\;\ln\left(\frac{\theta_{i}}{\theta_{i}+\theta_{j}}\right)+(n_{ij}-y_{ij})\ln\left(\frac{\theta_{j}}{\theta_{i}+\theta_{j}}\right)\right]$$ (3)

Eq (3) can be simplified to:

$$l(\theta )=\sum_{i\neq j}\left[y_{ij}(\ln\;\theta_{i}-\ln\left(\theta_{i}+\theta_{j}\right))+(n_{ij}-y_{ij})(\ln\;\theta_{j}-\ln\left(\theta_{i}+\theta_{j}\right))\right]$$ (4)

Thus, the log-likelihood function can be expressed as:

$$l(\theta )=\sum_{i\neq j}\left[y_{ij}\;\ln\;\theta_{i}+(n_{ij}-y_{ij})\ln\;\theta_{j}-n_{ij}\ln\left(\theta_{i}+\theta_{j}\right)\right]$$ (5)

Maximum likelihood estimation aims to find the parameter values *θ* that maximize the log-likelihood function. This involves taking the partial derivative of ℓ(*θ*) with respect to each *θ*_*i*_ and setting it to zero. The partial derivative is given by:

$$\frac{\partial l(\theta )}{\partial \theta_{i}}=\sum_{i\neq j}[\frac{y_{ij}}{\theta_{i}}-\frac{n_{ij}}{\theta_{i}+\theta_{j}}]+\sum_{j\neq i}\left[\frac{y_{ji}}{\theta_{i}}-\frac{n_{ji}}{\theta_{i}+\theta_{j}}\right]$$ (6)

Setting this partial derivative to zero yields the maximum likelihood estimates.

### 4.1 Experimental results

The paired comparison experiment involved 15 evaluators comparing each pair of five acceleration profiles (A, B, C, D, and E). The win matrix **W** was constructed to represent the number of times each profile was preferred over the others across all comparisons. The win matrix for the experimental 1 is shown in Table 2.

**Table 2:** Win matrix for acceleration profiles in Experiment 1.

	A	B	C	D	E
**A**	0	4	7	1	9
**B**	11	0	7	8	6
**C**	8	8	0	7	9
**D**	14	7	8	0	10
**E**	6	9	6	5	0

The Bradley-Terry model was applied to the win matrix to estimate the ability parameters (*θ*) of each acceleration profile. Profile A was chosen as the reference profile because it represents the ideal acceleration curve, which allows for easier comparison and visualization of the other profiles’ performance. To facilitate visualization, centered estimates were calculated. The model’s primary outputs include the estimated ability parameters, their standard errors, and 95% confidence intervals, which are summarized in Table 3.

**Table 3:** Bradley-Terry model estimates for Experiment 1.

Profile	Centered Estimate (*θ*)	Standard Error	95% CI Lower	95% CI Upper	Order
**A**	-0.5032	0.0000	-0.5032	-0.5032	5
**B**	0.1100	0.3418	-0.5599	0.7799	2.5
**C**	0.1100	0.3417	-0.5598	0.7797	2.5
**D**	0.5034	0.3497	-0.1820	1.1889	1
**E**	-0.2201	0.3419	-0.8903	0.4500	4

**Fig 9:**
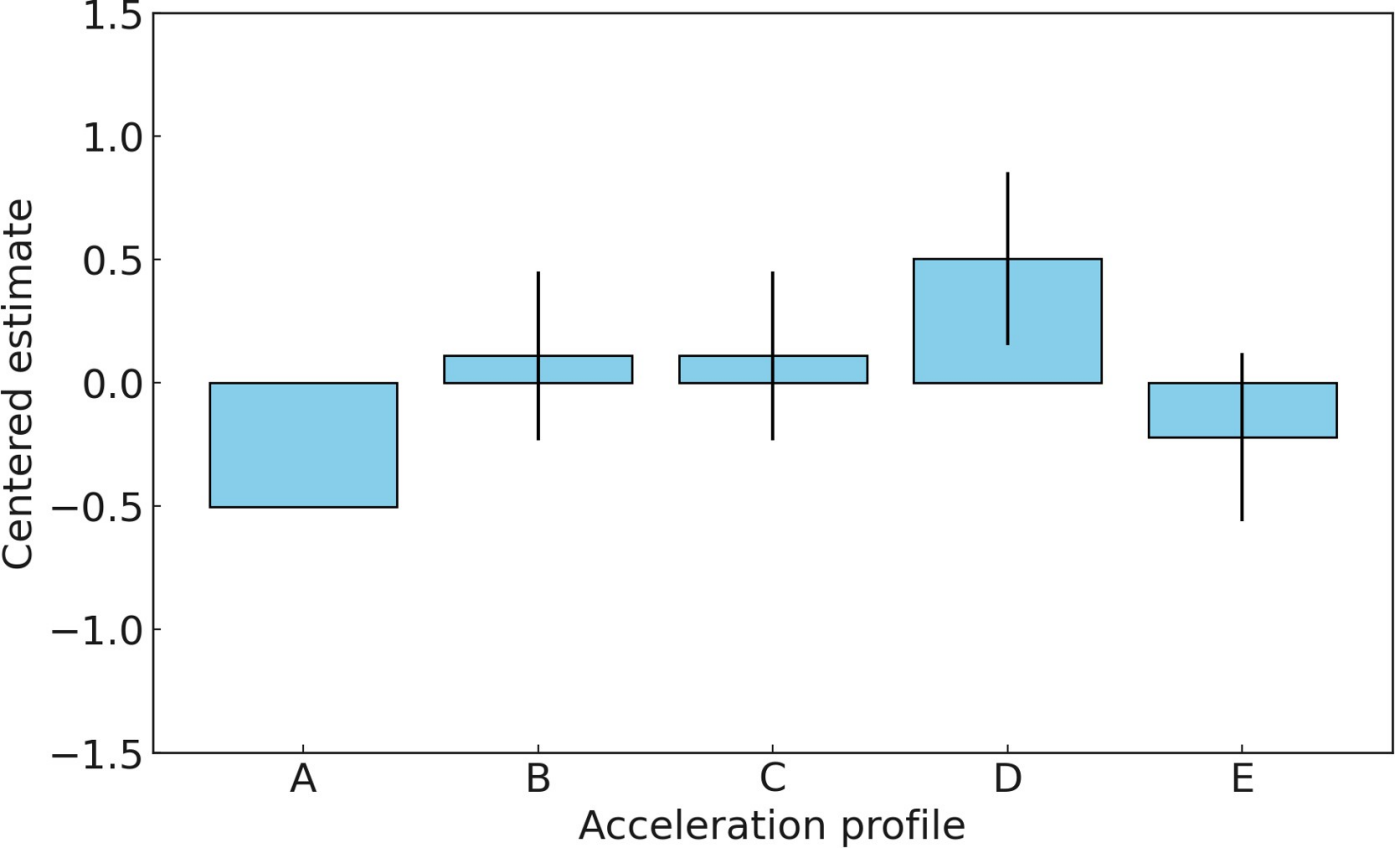
Estimated value for five acceleration profiles in Experiment 1.

The ability parameters indicate the relative preference for each acceleration profile. Higher values of *θ* correspond to higher preferences. Profile D has the highest estimated ability parameter (0.5034), making it the most preferred acceleration profile over all other profiles. Profile E is the second most preferred profile, with an estimated ability parameter of -0.2201. Profile C and Profile B both have estimated ability parameters of 0.1100, suggesting they are moderately preferred, but less so than Profile D and Profile E. Profile A has the lowest estimated ability parameter (-0.5032), indicating it is the least preferred acceleration profile among the evaluators. Despite Profile A being characterized by the fastest approach to the driver’s desired acceleration, it was the least preferred among the evaluators due to the experimental scenario involving low tip-in acceleration, where the driver’s intention to accelerate is minimal. Fig 9 presents the estimated ability parameters for each profile in Experiment 1, along with their corresponding standard errors.

Next, the analysis for Experiment 2 was conducted similarly to Experiment 1. Initially, the win matrix was constructed as displayed in Table 4, and the Bradley-Terry model was employed to calculate the estimates. The outcomes of the Bradley-Terry model application are summarized in Table 5.

**Table 4:** Win matrix for acceleration profiles in Experiment 2.

	A	B	C	D	E
**A**	0	3	10	3	10
**B**	12	0	11	6	14
**C**	5	4	0	3	6
**D**	12	9	12	0	8
**E**	5	1	9	7	0

**Table 5:** Bradley-Terry model estimates for Experiment 2.

Profile	Centered Estimate (*θ*)	Standard Error	95% CI Lower	95% CI Upper	Order
**A**	-0.2385	0.0000	-0.2385	-0.2385	3
**B**	0.7853	0.3551	0.0894	1.4813	1
**C**	-0.7260	0.3501	-1.4121	-0.0398	5
**D**	0.6561	0.3488	-0.0275	1.3398	2
**E**	-0.4771	0.3395	-1.1424	0.1883	4

**Fig 10:**
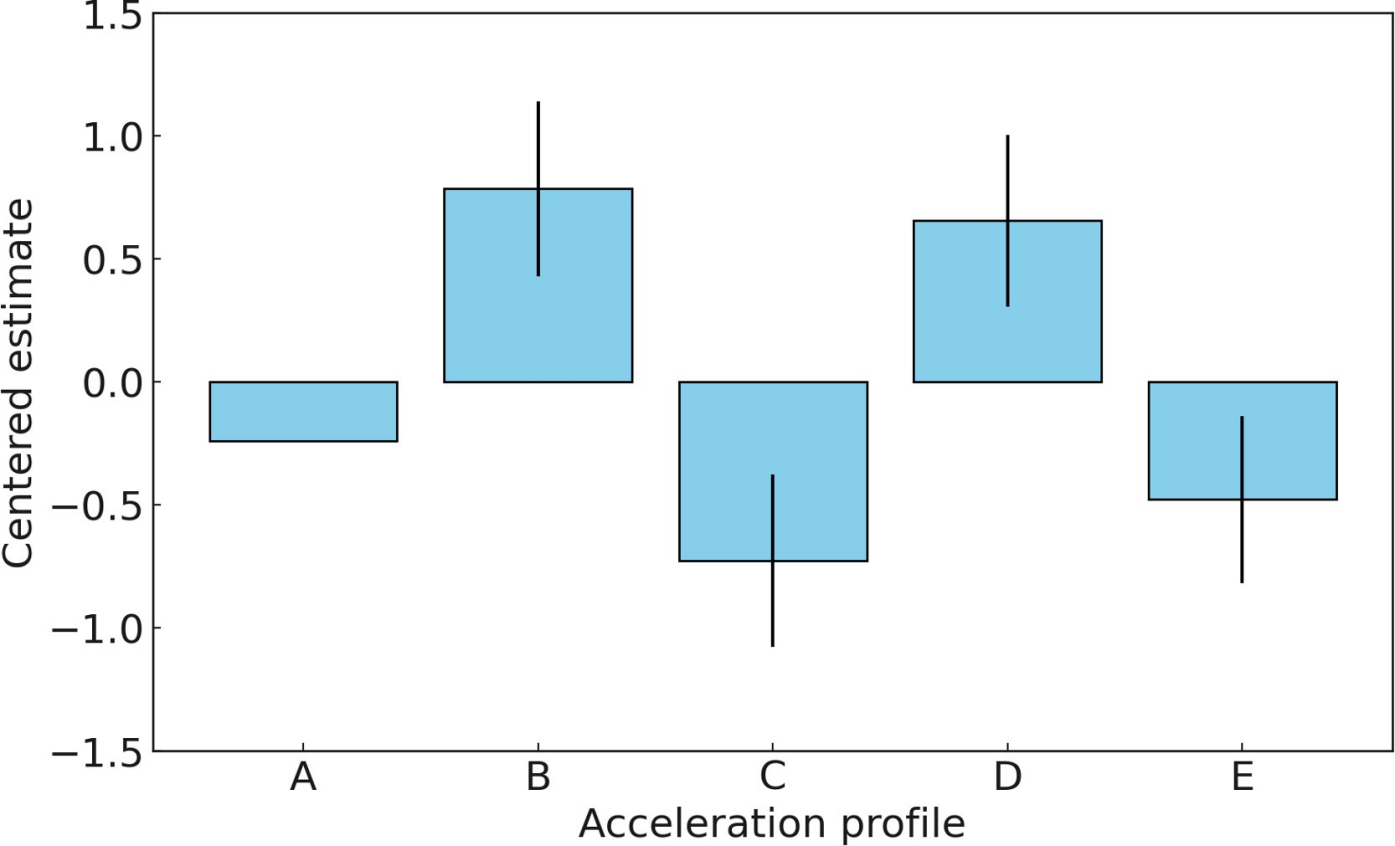
Estimated value for five acceleration profiles in Experiment 2.

In Experiment 2, Profile B emerged as the most favored acceleration profile with the highest estimated ability parameter (0.7853). Profile D was the next preferred, with an ability parameter of 0.6561. Profile A, with a centered estimate of -0.2385, ranked in the middle. Profile E had a centered estimate of -0.4771, making it less favored than Profile A, while Profile C had the lowest estimate (-0.7260), indicating the least preference among the evaluators in Experiment 2. Fig 10 illustrates the estimated ability parameters for each profile in Experiment 2, along with their standard error.

Lastly, the results of the analysis for Experiment 3 are presented. Tables 6 and 7 display the win matrix and the results of the Bradley-Terry model application, respectively.

**Table 6:** Win matrix for acceleration profiles in Experiment 3.

	A	B	C	D	E
**A**	0	7	11	9	14
**B**	8	0	8	7	11
**C**	4	7	0	7	8
**D**	6	8	8	0	15
**E**	1	4	7	0	0

**Table 7:** Bradley-Terry model estimates for Experiment 3.

Profile	Centered Estimate (*θ*)	Standard Error	95% CI Lower	95% CI Upper	Order
**A**	0.6734	0.0000	0.6734	0.6734	3
**B**	0.2509	0.3389	-0.4133	0.9151	1
**C**	-0.2185	0.3454	-0.8955	0.4585	5
**D**	0.4287	0.3373	-0.2324	1.0898	2
**E**	-1.1345	0.3910	-1.9009	-0.3681	4

**Fig 11:**
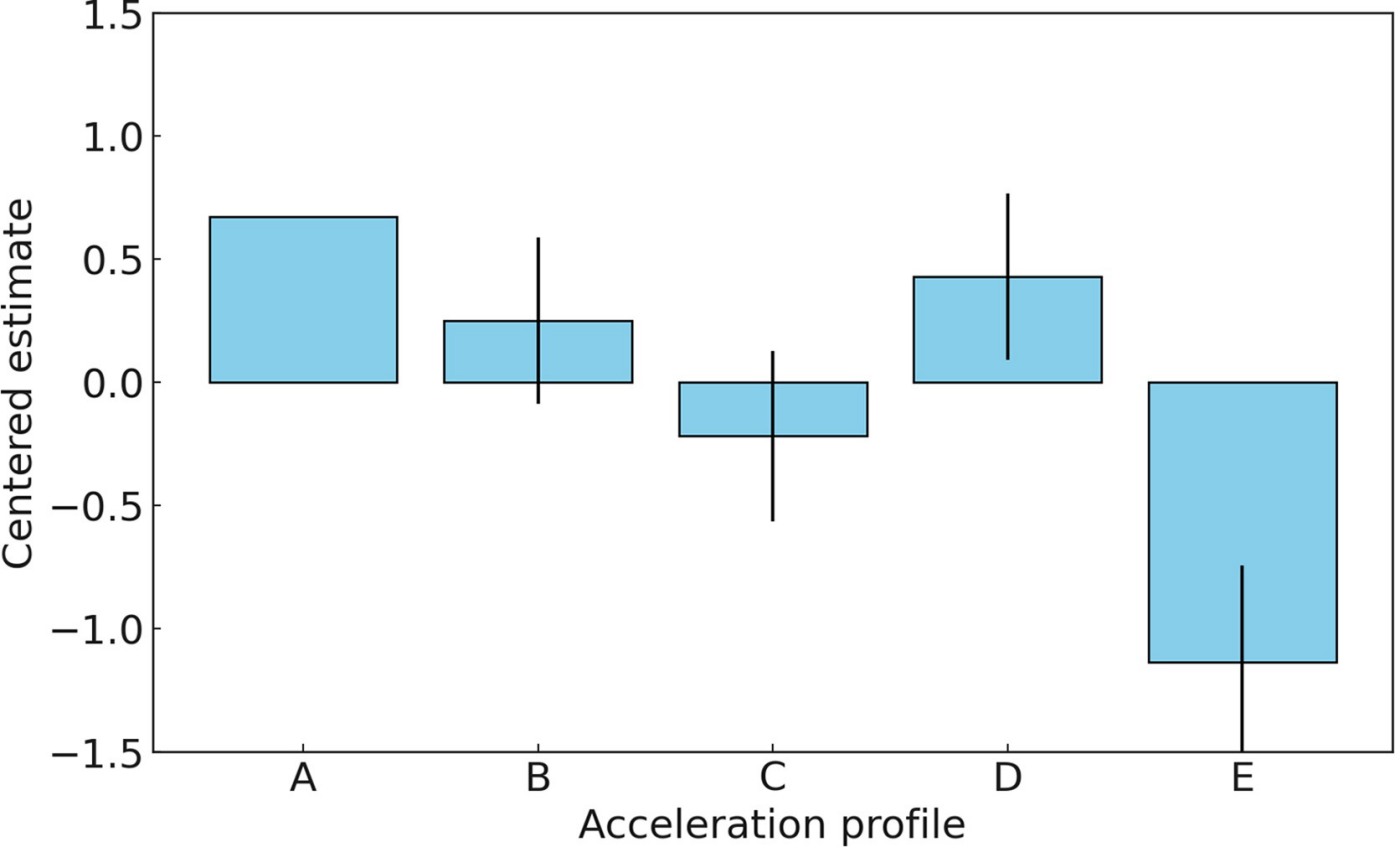
Estimated value for five acceleration profiles in Experiment 3.

In Experiment 3, Profile B is identified as the most favored acceleration profile with the highest estimated ability parameter (0.2509). Profile D follows closely with an ability parameter of 0.4287, indicating a high level of preference as well. Profile A, with a centered estimate of 0.6734, ranks third in preference. Profile E, having a centered estimate of -1.1345, is less favored than Profile A but ranks higher than Profile C, which has the lowest estimate (-0.2185), indicating it is the least preferred acceleration profile among the evaluators in Experiment 3. Fig 11 illustrates the estimated ability parameters for each profile in Experiment 3, along with their standard error.

### 4.2 Comparison with dynamic characteristics

To further explore the factors influencing driver preferences, we examined the dynamic characteristics of each acceleration profile, including maximum jerk, time to maximum jerk, acceleration gradient, and kurtosis of jerk. These characteristics were then compared to the Bradley-Terry model estimates to understand their impact. The definitions for the terms ’maximum jerk,’ ’time to maximum jerk,’ ’acceleration gradient,’ and ’kurtosis of jerk’ are as follows:

- Maximum jerk: Maximum jerk refers to the highest rate of change of acceleration experienced during a specific period. In other words, it is the peak value of the derivative of acceleration with respect to time. This measure is crucial in assessing the abruptness or smoothness of changes in acceleration, which can significantly affect driver comfort and vehicle control.
- Time to maximum jerk: Time to maximum jerk is the duration it takes from the start of the acceleration event to reach the maximum jerk. This measure helps in understanding the dynamics of acceleration changes and the temporal characteristics of how quickly a vehicle can reach its peak rate of change in acceleration.
- Acceleration gradient: The acceleration gradient represents the rate at which acceleration itself changes over time. It is the second derivative of position with respect to time or the first derivative of acceleration with respect to time. This parameter is essential for evaluating how quickly a vehicle can change its acceleration, impacting the smoothness of ride and handling.
- Kurtosis of jerk: Kurtosis of jerk is a statistical measure that describes the tailedness or peakedness of the jerk distribution over a period. High kurtosis indicates that the jerk profile has more extreme values, suggesting a higher likelihood of experiencing significant changes in acceleration. In contrast, low kurtosis implies a more uniform and less extreme distribution of jerk values. This measure is important for understanding the variability and extremity of acceleration changes, influencing both comfort and vehicle dynamics.

These definitions help elucidate the dynamic characteristics of acceleration profiles, which are crucial for understanding driver preferences and their alignment with the Bradley-Terry model estimates.

**Fig 12:**
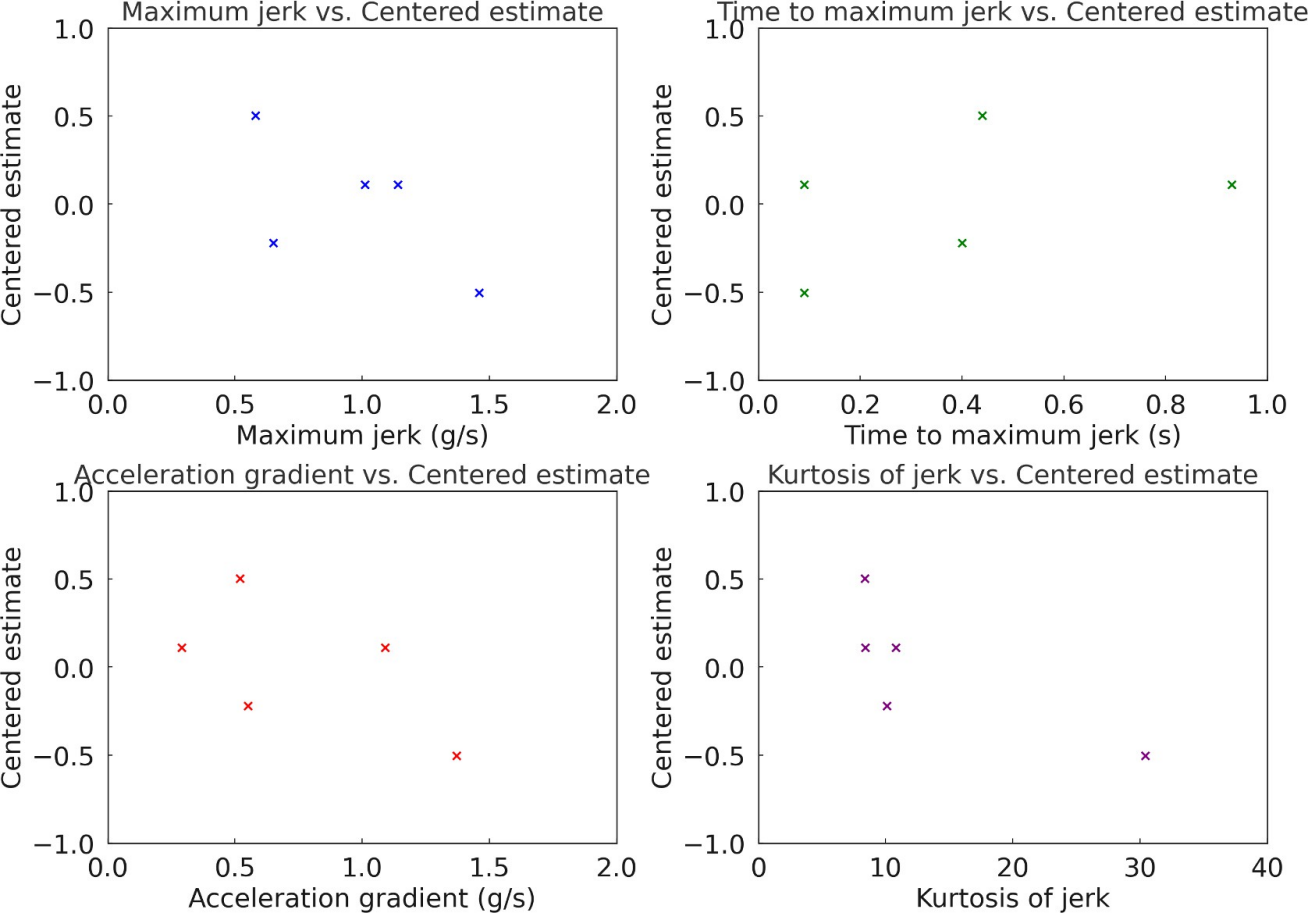
Correlation between dynamic characteristics of longitudinal acceleration and subjective preference in Experiment 1.

In Experiment 1, conducted at an initial vehicle speed of 30 km/h with low tip-in and a target acceleration of 0.10 g, the dynamic characteristics of each acceleration profile were analyzed to understand their influence on driver preferences. Table 8 shows the dynamic characteristics of each acceleration profiles in Experiment 1. Further, Fig 12 illustrates the correlation between dynamic characteristics and estimates in Experiment 1. The Pearson correlation coefficients between the estimate and the dynamic characteristics of maximum jerk, time to maximum jerk, acceleration gradient, and kurtosis of jerk were determined to be -0.6361, 0.3845, -0.5811, and -0.7788, respectively. The correlation analysis indicates that profiles with lower maximum jerk, more gradual acceleration gradients, and lower kurtosis of jerk are more favored by drivers. Specifically, the high negative correlations between maximum jerk (*r* = −0.6361) and kurtosis of jerk (*r* = −0.7788) with the estimate suggest that smoother profiles are preferred. This indicates that profiles which minimize abrupt changes in acceleration (low jerk and low kurtosis) tend to enhance driver comfort. Conversely, the positive correlation with time to maximum jerk (*r* = 0.3845) implies that profiles which take longer to reach peak jerk are also preferred, as these profiles likely provide a smoother transition to the desired acceleration. Profile D, representing a linear acceleration curve, emerged as the most preferred profile, while Profile A, characterized by the fastest approach to the desired acceleration, was the least preferred. The experimental results indicate that drivers at a low vehicle speed of 30 km/h favor acceleration profiles that offer a more gradual and linear increase in acceleration, avoiding abrupt changes. This preference for smoother acceleration patterns is consistent with the observed correlations, suggesting that enhancing driver preference requires minimizing abrupt jerks and providing gradual transitions in acceleration. These findings underscore the importance of designing EV acceleration profiles that prioritize smooth and gradual changes to improve driver satisfaction and comfort in low speed and low tip-in conditions.

**Table 8:** Dynamic characteristics of each acceleration profile in Experiment 1.

Profile	Maximum jerk (*g*/*s*)	Time to maximum jerk (s)	Acceleration gradient (*g*/*s*)	Kurtosis of jerk
A	1.46	0.09	1.37	30.39
B	1.01	0.09	1.09	10.81
C	1.14	0.93	0.29	8.38
D	0.58	0.44	0.52	8.37
E	0.65	0.40	0.55	10.10

**Fig 13:**
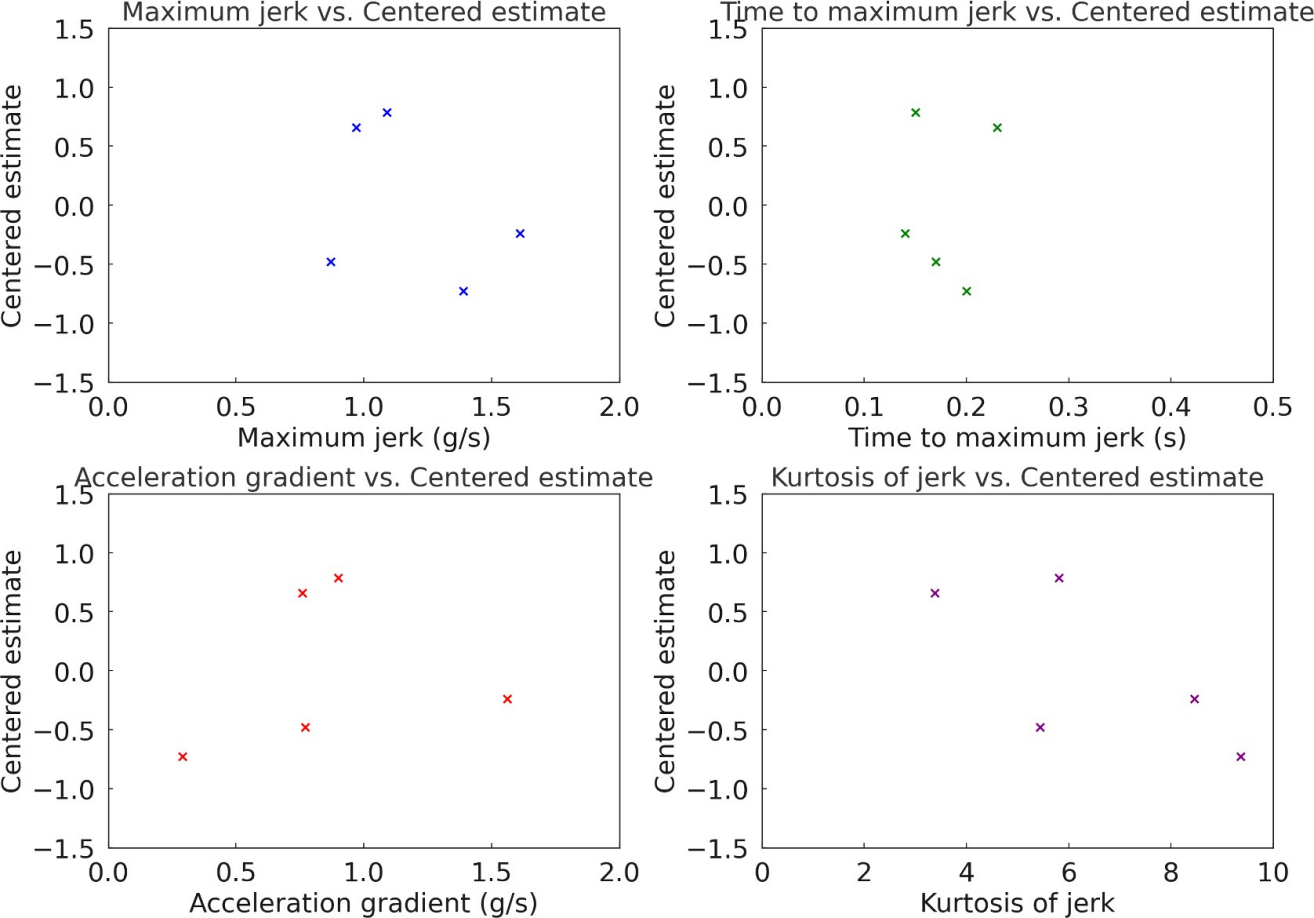
Correlation between dynamic characteristics of longitudinal acceleration and subjective preference in Experiment 2.

Subsequently, Table 9 shows the dynamic characteristics of each acceleration profile in Experiment 2, and Fig 13 illustrates the correlation between dynamic characteristics and estimates in Experiment 2. The Pearson correlation coefficients between the estimate and the dynamic characteristics of maximum jerk, time to maximum jerk, acceleration gradient, and kurtosis of jerk were determined to be -0.3796, 0.0895, 0.2031, and -0.7024, respectively. In Experiment 2, conducted at the same initial speed but with middle tip-in and a higher target acceleration of 0.15 g, Profile B, known for its rapid initial acceleration followed by a smooth progression, was the most favored. Profile D also remained significantly preferred, while Profile C, designed to mitigate shock from backlash, was the least favored. In other words, drivers favored acceleration profiles that exhibited a moderate initial acceleration phase followed by a gradual and consistent increase in acceleration. The correlation analysis for Experiment 2 reveals the following relationships between the dynamic characteristics and the estimated preferences: Maximum jerk shows a moderate negative correlation with the estimate (*r* = -0.3796), indicating that profiles with higher maximum jerk tend to be less preferred. Time to maximum jerk exhibits a weak positive correlation with the estimate (*r* = 0.0895), and acceleration gradient shows a weak positive correlation with the estimate (r = 0.2031), indicating a minimal preference for profiles with steeper acceleration gradients. Kurtosis of jerk exhibits a high negative correlation with the estimate (r = -0.7024), suggesting that profiles with higher kurtosis, implying more abrupt changes, are less preferred. The analysis indicates that in Experiment 2, the dynamic characteristics of maximum jerk and kurtosis of jerk significantly influence driver preferences. Specifically, profiles that exhibit lower maximum jerk and lower kurtosis are more preferred. The weak positive correlations with time to maximum jerk and acceleration gradient suggest that these characteristics have less impact on preferences under these experimental conditions. Given these results, it is recommended to design acceleration profiles that minimize abrupt changes and provide smoother transitions to improve driver preferences.

**Table 9:** Dynamic characteristics of each acceleration profile in Experiment 2.

Profile	Maximum jerk (*g*/*s*)	Time to maximum jerk (s)	Acceleration gradient (*g*/*s*)	Kurtosis of jerk
**A**	1.61	0.14	1.56	8.46
**B**	1.09	0.15	0.90	5.81
**C**	1.39	0.20	0.29	9.36
**D**	0.97	0.23	0.76	3.38
**E**	0.87	0.17	0.77	5.44

**Fig 14:**
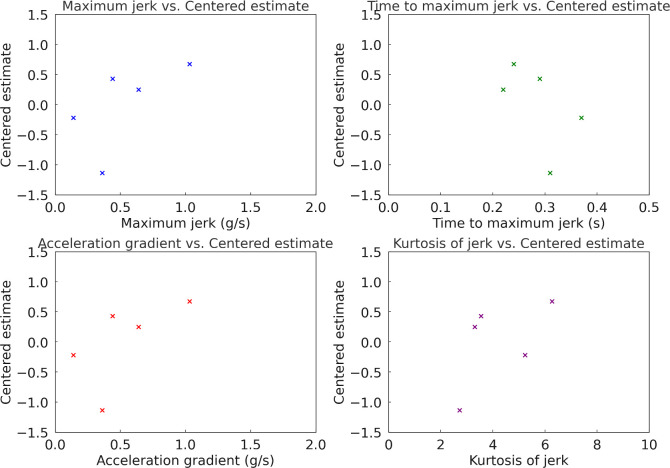
Correlation between dynamic characteristics of longitudinal acceleration and subjective preference in Experiment 3.

Lastly, Table 10 outlines the dynamic characteristics of each acceleration profile in Experiment 3. Fig 14 depicts the correlation between these dynamic characteristics and the estimates in Experiment 3. The Pearson correlation coefficients between the estimate and the dynamic characteristics of maximum jerk, time to maximum jerk, acceleration gradient, and kurtosis of jerk were found to be 0.78, -0.62, 0.78, and 0.57, respectively. In Experiment 3, conducted at a higher initial speed of 60 km/h with middle tip-in and a target acceleration of 0.10 g, Profile A became the most preferred. This profile, with the fastest approach to desired acceleration, showed its effectiveness under higher speed conditions. Profile D continued to show strong preference, while Profile E was the least favored in this scenario. Examining the correlation analysis for Experiment 3 uncovers the following relationships between the dynamic characteristics and the estimated preferences: Maximum jerk shows a high positive correlation with the Estimate (*r* = 0.6349), indicating that profiles with higher maximum jerk are more preferred. Time to maximum jerk exhibits a moderate negative correlation with the Estimate (*r* = −0.5388), suggesting that profiles with shorter times to reach maximum jerk are more preferred. Acceleration gradient displays a relatively high positive correlation with the estimate (*r* = 0.6349), pointing to a preference for profiles with steeper acceleration gradients. Kurtosis of jerk presents a moderate positive correlation with the estimate (*r* = 0.5527), suggesting that profiles with higher kurtosis are more preferred. These findings demonstrate that in Experiment 3, the dynamic characteristics of maximum jerk, time to maximum jerk, acceleration gradient, and kurtosis of jerk significantly influence driver preferences. Specifically, profiles that exhibit higher maximum jerk, shorter times to reach maximum jerk, and steeper acceleration gradients are more preferred. The moderate positive correlation with kurtosis of jerk indicates that drivers also favor profiles with more abrupt changes. Based on these insights, it is recommended to design acceleration profiles that incorporate higher maximum jerk and steeper acceleration gradients while ensuring quick transitions to the desired acceleration to improve driver preferences.

**Table 10:** Dynamic characteristics of each acceleration profile in Experiment 3.

Profile	Maximum jerk (*g*/*s*)	Time to maximum jerk (s)	Acceleration gradient (*g*/*s*)	Kurtosis of jerk
**A**	1.03	0.24	1.03	6.27
**B**	0.64	0.22	0.64	3.31
**C**	0.14	0.37	0.14	5.24
**D**	0.44	0.29	0.44	3.55
**E**	0.36	0.31	0.36	2.73

### 4.3 Discussion

Across all three experiments, it is evident that driver preferences for acceleration profiles are influenced by the dynamic characteristics of those profiles, with the specific impacts varying based on the driving conditions. At lower speeds and lower target accelerations (Experiment 1), smoother profiles with minimal abrupt changes are favored, emphasizing the importance of comfort and gradual transitions. As the target acceleration increases (Experiment 2), the preference for smooth profiles persists, but other characteristics like response time and gradient start to play a role. At higher speeds (Experiment 3), the preference shifts towards profiles that offer quick, dynamic responses, with higher jerk and steeper gradients being more desirable. Notably, the preference for linear acceleration profiles with smaller jerk magnitudes at 30 km/h suggests a high value placed on comfort and predictability during urban driving scenarios characterized by lower speeds. In contrast, the preference for more rapid acceleration profiles at higher speeds can be interpreted as a desire for enhanced responsiveness and performance during more dynamic driving conditions, such as highway travel. These findings are consistent with previous studies that have demonstrated the perceptual characteristics of vehicle acceleration vary between different acceleration conditions [38]. This suggests that basing the design of vehicle acceleration profiles on human perception and subjective preferences is an appropriate approach. These findings have significant implications for the design of EV acceleration profiles. To enhance driver satisfaction and comfort, it is crucial to tailor acceleration profiles to specific driving conditions. For urban and low-speed driving, profiles that prioritize smoothness and gradual transitions are ideal. For higher-speed scenarios, profiles that offer rapid, dynamic responses with higher jerk and steeper gradients are more likely to be preferred.

While the study provides practical insights, it is not without limitations. The scope of the experimental conditions, though comprehensive within its setup, does not encompass the full spectrum of real-world driving scenarios. Future research could expand on this by exploring a broader variety of driving conditions, including varying road types, weather conditions, and traffic patterns, which could influence acceleration preferences. Additionally, another limitation is the homogeneous demographic profile of the participants. The study predominantly involved highly experienced male drivability experts, which may not reflect the preferences of the general driving population. Future research should aim to include a diverse participant pool, incorporating various age groups, genders, driving experiences, and cultural backgrounds. This approach would ensure that the findings are more generalizable and applicable to a broader audience, enhancing the validity of the recommendations for EV acceleration profile designs. Further, another limitation is the controlled environment in which the experiments were conducted. Real-world driving involves dynamic and unpredictable elements that may affect driver preferences differently compared to controlled conditions. Incorporating on-road testing in diverse environments could enhance the ecological validity of the findings. Moreover, the study primarily focuses on acceleration profiles, but other aspects of drivability, such as braking and handling, are also crucial for overall vehicle performance and driver satisfaction. Future studies should consider a more holistic approach by integrating these additional aspects to provide a more comprehensive understanding of drivability preferences. Investigating the interplay between acceleration, braking, and handling characteristics could lead to more refined and balanced vehicle dynamics that enhance overall driver experience. Furthermore, the technological aspects of EVs, such as battery performance, energy efficiency, and regenerative braking, were not considered in this study. Future research should explore how these factors interplay with acceleration preferences, potentially leading to optimized designs that balance driver satisfaction with technical efficiency. Understanding how acceleration profiles impact energy consumption and battery life could help in developing strategies that optimize both performance and sustainability. Moreover, this study does not account for variations in vehicle load, which can significantly impact acceleration characteristics. Future research should incorporate different load conditions to better understand their influence on driver preferences and vehicle performance.

By addressing these limitations and expanding the scope of future research, we can develop a deeper and more nuanced understanding of driver preferences, ultimately leading to the design of EVs that better meet the needs and expectations of a diverse range of drivers in various real-world scenarios. This comprehensive approach will contribute to the advancement of EV technology and its adoption, aligning with the broader goals of sustainable and user-centric transportation solutions.

## 5. Conclusion

This study systematically examined driver preferences for different acceleration profiles in EVs under three distinct experimental conditions, using subjective evaluations obtained from on-road tests. Our aim was to provide design guidance for longitudinal acceleration profiles that align with driver preferences, enhancing the effectiveness of drivability development strategies. At a low initial vehicle speed of 30 km/h with low tip-in and a target acceleration of 0.10 g, the results revealed a clear preference for smoother acceleration profiles. Drivers favored linear acceleration profiles characterized by smaller jerk magnitudes and lower kurtosis values, emphasizing the importance of comfort and gradual transitions in low-speed urban driving scenarios. When the initial vehicle speed remained at 30 km/h but with middle tip-in and a higher target acceleration of 0.15 g, the preference for smooth profiles persisted. However, the findings indicated that drivers also valued quicker responses and moderate gradients, suggesting a preference for rapid initial acceleration followed by a smooth transition to the target acceleration. This reflects a nuanced balance between comfort and performance at moderate acceleration levels. At a higher initial vehicle speed of 60 km/h with medium tip-in and a target acceleration of 0.10 g, there was a distinct shift in driver preferences. Drivers expressed a preference for profiles characterized by greater jerk magnitudes and steeper acceleration gradients, highlighting the importance of responsiveness and dynamic performance in high-speed driving scenarios. This suggests that at higher speeds, drivers prioritize quick and dynamic responses over smoothness. The correlation analysis across all three experiments provided insights into the relationship between subjective preferences and the dynamic characteristics of the acceleration profiles. Specific characteristics such as maximum jerk, time to peak jerk, acceleration gradient, and kurtosis of jerk were found to influence driver preferences to varying degrees under different driving conditions. Notably, smoother profiles with minimal abrupt changes were preferred at lower speeds, while more rapid and dynamic profiles were favored at higher speeds. This study has limitations. The controlled conditions do not cover all real-world driving scenarios. Future research should explore varied conditions, including different roads, weather, and traffic patterns. Involving a more diverse group of participants will provide a broader understanding of driver preferences. Additionally, this study focused on acceleration profiles. Future research should also examine braking and handling to offer a more comprehensive view of vehicle performance and driver satisfaction. In conclusion, the findings of this study contribute to the field of EV development by offering practical guidance for designing acceleration profiles that align with driver preferences. By incorporating these preferences into the design process, manufacturers can enhance the drivability and overall user experience of their vehicles, potentially leading to higher customer satisfaction and increased adoption rates. As the automotive industry continues to shift towards electrification, understanding and meeting driver expectations will be crucial for market success. Future research should aim to validate these findings across diverse driving conditions and populations, incorporating real-time feedback to optimize EV designs further.

## Supporting information

S1 File Experiment data. (CSV)
