# Correction: Toward better drivability: Investigating user preferences for tip-in acceleration profiles in electric vehicles

**DOI:** 10.1371/journal.pone.0321684

**Published:** 2025-04-01

**Authors:** The PLOS One Staff

In the Experimental results, equations three through six are missing the function name ‘l’. Please view the complete, correct equation here:

$$l\left(\theta \right)=\ln L\left(\theta \right)=\sum_{i\neq j}\left[y_{ij}\ln\left(\frac{\theta_{i}}{\theta_{i}+\theta_{j}}\right)+\left(n_{ij}-y_{ij}\right)\ln\left(\frac{\theta_{j}}{\theta_{i}+\theta_{j}}\right)\right]$$ (3)

$$l\left(\theta \right)=\sum_{i\neq j}\left[y_{ij}\left(\ln\theta_{i}-\ln\left(\theta_{i}+\theta_{j}\right)\right)+\left(n_{ij}-y_{ij}\right)\left(\ln\theta_{j}-\ln\left(\theta_{i}+\theta_{j}\right)\right)\right]$$ (4)

$$l\left(\theta \right)=\sum_{i\neq j}\left[y_{ij}\ln\theta_{i}+\left(n_{ij}-y_{ij}\right)\ln\theta_{j}-n_{ij}\ln\left(\theta_{i}+\theta_{j}\right)\right]$$ (5)

$$\frac{\partial l\left(\theta \right)}{\partial \theta_{i}}=\sum_{i\neq j}\left[\frac{y_{ij}}{\theta_{i}}-\frac{n_{ij}}{\theta_{i}+\theta_{j}}\right]+\sum_{j\neq i}\left[\frac{y_{ji}}{\theta_{i}}-\frac{n_{ji}}{\theta_{i}+\theta_{j}}\right]$$ (6)

The publisher apologizes for the error.

## References

[1] Kim S, Yang J. Toward better drivability: Investigating user preferences for tip-in acceleration profiles in electric vehicles. PLoS One. 2024;19(12): e0311504. doi: 10.1371/journal.pone.0311504 39666752 PMC11637387

